# Heterogeneity of prostate-specific membrane antigen (PSMA) and PSMA-ligand uptake detection combining autoradiography and postoperative pathology in primary prostate cancer

**DOI:** 10.1186/s13550-023-01044-8

**Published:** 2023-11-16

**Authors:** Hui Wang, Marianne Remke, Thomas Horn, Kristina Schwamborn, Yiyao Chen, Katja Steiger, Wilko Weichert, Hans-Jürgen Wester, Margret Schottelius, Wolfgang A. Weber, Matthias Eiber

**Affiliations:** 1 Department of Nuclear Medicine, West China Hospital, Sichuan University, Guo Xue Xiang 37, Chengdu, 610040 Sichuan China; 2 Department of Nuclear Medicine, Klinikum Rechts der Isar, Technical University Munich, Ismaninger Str. 22, 81675 Munich, Germany; 3 Institute of Pathology, School of Medicine, Technical University Munich, Munich, Germany; 4 Department of Urology, Klinikum Rechts der Isar, Technical University Munich, Munich, Germany; 5 Departments of Mathematics and Life Sciences, Technical University Munich, Munich, Germany; 6 German Cancer Consortium (DKTK), Heidelberg, Germany; 7 Pharmaceutical Radiochemistry, Technical University Munich, Munich, Germany; 8 Translational Radiopharmaceutical Sciences, Departments of Nuclear Medicine and of Oncology, Centre Hospitalier Universitaire Vaudois, University of Lausanne, Lausanne, Switzerland; 9 Agora, Pole de Recherche Sur Le Cancer, Lausanne, Switzerland

**Keywords:** Primary prostate cancer, Heterogeneity, ^99m^Tc-PSMA-I&S, ARG, Immunohistochemistry

## Abstract

**Background:**

Targeting prostate-specific membrane antigen (PSMA) has been highly successful for imaging and treatment of prostate cancer. However, heterogeneity in immunohistochemistry indicates limitations in the effect of imaging and radionuclide therapy of multifocal disease. ^99m^Tc-PSMA-I&S is a γ-emitting probe, which can be used for intraoperative lesion detection and postsurgical autoradiography (ARG). We aimed to study its intraprostatic distribution and compared it with (immuno)-histopathology.

**Results:**

Seventeen patients who underwent RGS between 11/2018 and 01/2020 with a total of 4660 grids were included in the preliminary analysis. Marked intratumor and intra-patient heterogeneity of PSMA expression was detected, and PSMA negative foci were observed in all samples (100%). Heterogeneous intra-patient PSMA-ligand uptake was observed, and no significant correlation was present between the degree of heterogeneity of PSMA expression and PSMA-ligand uptake. Higher PSMA-ligand uptake was observed in GS ≥ 8 than GS < 8 (*p* < 0.001). The appearance of Gleason Pattern (GP) 4 was strongly associated with higher uptake (coefficient: 0.43, *p* < 0.001), while GP 5 also affected the uptake (coefficient: 0.07, *p* < 0.001).

**Conclusion:**

PSMA expression and PSMA-ligand uptake show marked heterogeneity. Prostate carcinoma with GP 4 showed significantly higher uptake compared with non-neoplastic prostate tissue. Our analyses extend the scope of applications of radiolabeled PSMA-ligands to ARG for identifying high-grade disease and using its signal as a noninvasive biomarker in prostate cancer.

**Supplementary Information:**

The online version contains supplementary material available at 10.1186/s13550-023-01044-8.

## Introduction

Prostate cancer is the second most common cancer in men worldwide [1, 2]. In the majority of cases, it overexpresses the prostate-specific membrane antigen (PSMA) which is a type II transmembrane glycoprotein with folate hydrolase activity [3, 4]. Early studies associated PSMA expression with prostate cancer malignancy, and a positive correlation between PSMA expression and Gleason Pattern (GP) was observed [3, 5–8]. In particular, relatively low expression of PSMA was found in Gleason Pattern (GP) 3 compared with GP 4 and GP 5 (*p* < 0.001) [9]. Moreover, PSMA expression can be heterogeneous even within the same primary tumor [10], and around 5–10% of primary prostate cancers are PSMA negative on immunohistochemistry (IHC) [11]. Increasing evidence shows that the level of PSMA expression of the primary tumor is associated with a worse prognosis [11–15].

The use of positron emission tomography (PET) probes targeting PSMA has gained increasing interest for both imaging and therapy. It offers superb specificity for prostate tissues and provides excellent contrast-to-noise ratio improving the detectability of lesions [16, 17]. However, the increasing body of clinical evidence also outlines substantial limitations: For primary lymph node staging, different information on sensitivity of PSMA-Ligand-PET is found in the literature [18–22], and PSMA-negativity of the primary tumor in PET has also been reported [23]. Screening for the VISION trial using ^177^Lu-PSMA-617 radioligand therapy (RLT) in metastatic castration-resistant prostate cancer excluded approximately 12% of patients due to missing or low PSMA expression of known tumor lesions in ^68^ Ga-PSMA11-PET [24]. In addition, a substantial number of eligible patients shows inadequate response to PSMA-targeted RLT [25–27] which among other factors might be attributed to heterogeneity of intralesional PSMA expression.

PSMA IHC has been extensively used in prostate cancer to validate and describe PSMA expression using both antibodies targeting the extra- or intracellular domain of PSMA [28]. For example, the J591 PSMA antibody binds a site located in the apical region of the extracellular domain of PSMA. However, PSMA-ligands used in the clinics both for diagnostic and therapeutic procedures are small molecules and bind the enzymatic pocket of PSMA based on the glutamate-urea-lysine moiety [29] [30–32]. A variety of different agents (e.g., PSMA-11, PSMA-617, PSMA I&T, rhPSMA-7.3) have been developed with different linkers and chelators and are in clinical evaluation.

In 2016, ^99m^Tc-PSMA-I&S (imaging and surgery) was developed, a ^99m^Tc-labeled probe for PSMA-targeted radioguided surgery (PSMA-RGS) with high stability in vivo and elevated lesion-to-background contrast at the time of surgery [33–35]. PSMA-RGS allows intraoperative identification of metastatic lesions by visual and acoustic feedback and is mainly used to improve surgical precision during salvage lymph adenectomy [36]. Currently, PSMA-RGS is also expanded to the setting of primary prostate cancer surgery, especially when tiny and atypical located lymph node metastasis are present [37]. The surgically removed specimen still contains ^99m^Tc-PSMA-I&S whose radioactivity signal can be measured using autoradiography (ARG).

This offers the potential to directly compare the localization of the radioactive signals with histopathological findings. Performing ARG on prostate tissue that has already been cut for histopathological examination, avoids potential misalignment caused by manual image rotation and processing as usual for image co-registration of PET and histopathology. Until now, the intralesional distribution of PSMA-ligands in primary prostate cancer in direct comparison to histopathology has not been investigated [14]. We therefore, in this manuscript, present a detailed comparison of ^99m^Tc-PSMA-I&S uptake by high-resolution ARG, histopathology and IHC to assess the intraprostatic distribution of ^99m^Tc-PSMA-I&S on a microscopic level.

## Materials and methods

### Patients

Data from 17 patients who underwent ^99m^Tc-PSMA-RGS for primary prostate cancer with lymph node metastases between November 2018 and January 2020 in our institution were retrospectively analyzed. Patients with other treatment before the surgery or distant metastases were excluded. Patient characteristics are provided in Table 1. The surgically removed prostates underwent standard histopathological evaluation including additional ARG to provide ancillary information on intraprostatic tumor extent. The retrospective analysis was approved by the Ethics Committee of the Technical University Munich (750/20 S-KH).

**Table 1:** Patient characteristics

Characteristic	n
No. of patients	17
Age (y), median (IQR)	69 (64.5–72)
PSA (ng/mL), median (IQR)	12.9 (7.23–28.37)
Injected activity ^99m^Tc-PSMA I&S, median (IQR)	678 (531–740)
ISUP grade*, no. (%)	
2	1 (5.9%)
3	6 (35.3%)
4	2 (11.8%)
5	7 (41.2%)
Pathological stage*, no. (%)	
pT status	
2c	4 (23.5%)
3a	2 (11.8%)
3b	9 (52.9%)
4	1 (5.9%)
pN status	
0	2 (11.8%)
1	14 (82.4%)

(M. R.) for sections in question

IQR,  interquartile range; ISUP,  International Society of Urologic Pathologists; PIN, Prostatic intraepithelial neoplasia; PSA, prostate-specific antigen

^*^In one of 17 patients, no information concerning Gleason Score and pathological stage could be obtained because the patient was reported high-grade PIN

### Histopathology, immunohistochemistry and autoradiography

To compare signal from autoradiography, histopathology and immunohistochemistry slices from routine pathology were used and processed / imaged using the following approach:

#### Sample preparation

After resection, the prostate was transferred to the Institute of Pathology and the specimen was cut perpendicular to the long axis of the urethra into 6–10 slides of 5–7 mm according to the diagnostic standard. Every second slice was put into a formalin-filled plastic bag filled with 4% neutral-buffered formalin (Additional file 1: Fig. 1).

#### Autoradiography

Samples were placed in a light-proof ARG cassette (Molecular Dynamics Storage Phosphor Screen, GE Healthcare, Chicago, United States) together with calibration standards allowing quantitative analyses. Photos of samples and calibration standards were taken prior to start of film exposure as references for image registration. Samples were exposed for 24 h in a dark enclosure to prevent exposure to ambient light (Additional file 1: Fig. 2). A phosphor-imaging plates (BAS-MS 2025; Fujifilm, Tokyo, Japan) was used and scanned on a CR35 Bio plate reader (Elysia-raytest, Straubenhardt, Germany) at 50 μm pixel resolution. Images were then analyzed using AIDA Image analyzer software (Version 4.21). The following procedure was performed to produce calibration standards in order to quantitatively assess the accumulation of ^99m^Tc-PSMA-I&S in the probes a calibration standard was introduced: A shape modified Grace Bio-Labs Press-To-Seal silicone isolator (Sigma-Aldrich, Missouri, United States) was fixed on a microscope slide. Ten 1:2 dilutions of ^99m^Tc-PSMA-I&S solution using DPBS (Gibco, Thermo Fisher Scientific, Waltham, USA) with increasing dilution were prepared. Five µl of each dilution were measured by a CRC-15R dose calibrator (Capintec, Inc, Florham Park, USA) and spotted into the tiny holes within the silicone. After final drying on the surface of a thermomixer (Eppendorf, Hamburg, Germany) they were placed into the cassette.

#### Histopathology and immunohistochemistry

Specimens were formalin fixed (at least 24 h in 4% neutral-buffered formalin solution) and paraffin embedded (FFPE). All FFPE samples were processed by the Comparative Experimental Pathology (CEP) unit at the Institute of Pathology, Technical University of Munich (TUM), Munich, Germany. Serial sections of 2 µm were cut using a rotary microtome (RM2245 Leica Biosystems, Wetzlar, Germany) for HE and IHC staining. IHC was performed automatically using the Ventana BenchMark XT (Roche, Basel, Switzerland). PSMA-Staining sections were pre-treated with Ventana Cell Conditioner 1 immunostainer (Ventana Medical Systems, Oro Valley, AZ, USA) for 20 min and incubated with mouse anti-PSMA (Dako M3620, Clone 3E6, dilution 1:50) for 32 min at room temperature. For visualization, we used the ultraView Universal DAB Detection Kit (Ventana Medical Systems, Oro Valley, AZ, USA). HE and IHC sections were scanned in 40 × magnification using a whole-slide brightfield scanner (Aperio CS scannerLeica Biosystems, Wetzlar, Germany) and were analyzed with the ImageScope Software (Version 12.4.0.7018).

### Image analysis

#### Image registration

Digitalized HE slides and autoradiographic data were co-registered using Adobe Photoshop (Version CS5). A combination of visual landmarks including tumor edges, holes from vessels, and ink marks were used to register image datasets.

#### Levels of analysis

*Patient-based analysis.* One slice of each patient’s prostate specimen (*n = *16, one was excluded because only prostatic intraepithelial neoplasia [PIN] and no invasive carcinoma was detected) was used for heterogeneity analysis including ARG, HE and PSMA IHC.

*Region of interest (ROI)-based analysis.* A ROI was defined in HE as a cancer cell containing area. To avoid spillover from adjacent cancer area at least 5 mm normal prostate tissue had to be present between different ROIs. ROIs were generated manually in the HE staining using the ImageScope Software (Version 12.4.0.7018) and the same ROIs were applied to the corresponding ARG data.

*Grid-based analysis.* A 3 × 3 mm^2^ matrix was overlayed on each HE digital image. An experienced uro-pathologist (M. R.) and a board certified pathologist (K. S.) annotated the percentage of different tissue types (cancer cell [GP3, GP4 and GP5], normal epithelium, PIN, Stroma, Inflammation, seminal vesicle, and open area) within every 3 × 3 mm^2^ section and the same matrix was applied to the corresponding ARG data.

*Immunohistochemistry.* A four-point immunoreactive score (IRS) classification (Table 2) was used considering grade of membranous staining intensity (0 to 3) and percentage of positive cells. Heterogeneity was defined by different staining patterns existing in at least 5% of the studied region [38–40]. The slice, ROI and the 3 × 3 mm^2^ matrix were used as a fundamental unit of patient-, ROI-, and grid-based evaluation. Analyses were performed by one experienced investigator (H. W.) with support of an experienced pathologist.

**Table 2:** Immunoreactive and autoradiographic-reactive sore

Immunoreactive score (IRS) *		
Intensity of staining (membrane)	Percentage of positive cells	IRS^#^
0 = no color reaction	0 = no positive cells	0–1 = negative
1 = mild reaction	1 = < 10% positive cells	2–3 = mild
2 = moderate reaction	2 = 10%–50% positive cells	4–8 = moderate
3 = intense reaction	3 = 51%–80% positive cells	9–12 = strong
	4 = > 80%positive cells	

Autoradiographic-reactive score (ARS)		
Intensity of signal	Percentage of positive grids	ARS^§^
0 = < 0.5*average SUV of non-tumor grids	0 = no positive grids	0–1 = negative
1 = 1st tri-sectional quantile of the rest SUV	1 = < 10% positive grids	2–3 = mild
2 = 2nd tri-sectional quantile of the rest SUV	2 = 10%–50% positive grids	4–8 = moderate
3 = 3rd tri-sectional quantile of the rest SUV	3 = 51%–80% positive grids	9–12 = strong
	4 = > 80%positive cells	

^*^Modified from Woythal and Kaemmerer et al. (*41, 42*)

^#^$$\mathrm{IRS}=\mathrm{Intensity of staining}\times \mathrm{Percentage of positive cells}$$

^§^ $\mathrm{ARS}=\mathrm{Intensity of signal}\times \mathrm{Percentage of positive grids}$. Please note that the ARS is based on grids whereas the IRS takes into account the percentage of positive cells

#### Analysis of autoradiography

In patient-based analysis, ARG activity was evaluated using an autoradiographic-reactive score (ARS) system, which was developed to evaluate the degree of heterogenous PSMA-ligand uptake and included intensity of the ARG signal (0 to 3) and percentage of positive cells in an area (for details see Table 2).

For ROI-based analysis, ARG signal positivity was assessed visually. The positive signal was defined as a signal with higher uptake compared with normal prostatic tissue or background.

For grid-based analysis the respective SUV_ARG_ of every grid was calculated as follows: The quantum level (QL) as non-calibrated quantitative measure was extracted using the AIDA Image analyzer (Version 4.21) from every autoradiographic image on grid- base. The absolute radioactivity *A*_spec_ was assessed on the base of calibration curves (QL vs. *A*_spec_) generated using the respective calibration standards.

The SUV_ARG_ was calculated using the following formula:

$${\mathrm{SUV}}_{\mathrm{ARG}}=\frac{{\mathrm{A}}_{\mathrm{spec}}/{\mathrm{W}}_{\mathrm{spec}}}{{\mathrm{A}}_{\mathrm{inj}}/\mathrm{BWt}}$$

where *A*_spec_ is the specimen’s activity of the respective area decay corrected to the injection time point. *W*_spec_ is the grid’s weight. *A*_inj_ is the injected activity, and *BWt* is the weight of the patient.

$${\mathrm{W}}_{\mathrm{spec}}=3\times 3\times \frac{{\mathrm{L}}_{\mathrm{pros}}}{\mathrm{No}.\mathrm{ tiss}}\times {\uprho }_{\mathrm{pros}}$$

where *L*_pros_ is the length of the prostate from apex to base,* ρ*_pros_ (0.98 g/mL) is the density of prostate tissue [41]. *No*_tiss_ is the number of slices of the prostate specimen.

### Statistical analyses

Descriptive statistics were used to display continuous variables as the median and interquartile range (IQR) with 25th and 75th percentiles (Q1–Q3), mean ± standard deviation (SD), as well as percentages.

Continuous variables were compared using the unpaired Mann–Whitney test. The efficacy to predict prostate cancer using SUV_ARG_ was evaluated by the receiver operating characteristic (ROC) curve and the area under the curve (AUC). ANOVA was used to compare the means of more than three groups. The linear mixed model method was used for assessing the correlation between PSMA-ligand uptake and different tissue types, which could further provide the weight of each parameter.

To assess for intra-patient heterogeneity the Shannon–Wiener Index was calculated for IHC and PSMA-ligand uptake. The Shannon–Wiener Index equally weights positive areas and intensity of staining or signal to quantify heterogeneity [42]. The minimal Shannon–Wiener Index is 0, which represents homogeneity. The Shannon–Wiener Index was calculated for IHC and PSMA-ligand uptake. The Pearson correlation coefficient (r) was used for measuring association between two variables.

A *p* value less than 0.05 indicated statistical significance. All tests were two-tailed. Statistical evaluation was performed using SPSS (Version 20); graphs were generated using GraphPad Prism v8.

## Results

### Patient characteristics and ARG imaging

Histopathological and ARG data from 17 PC patients were analyzed. Gleason Score 7a, 7b, 8 and 9 were present in 1 (5.9%), 6 (35.3%), 2 (11.8%) and 7 (41.2%) patients, respectively. Postoperative pT and pN stages are provided in Table 1.

**Fig. 1:**
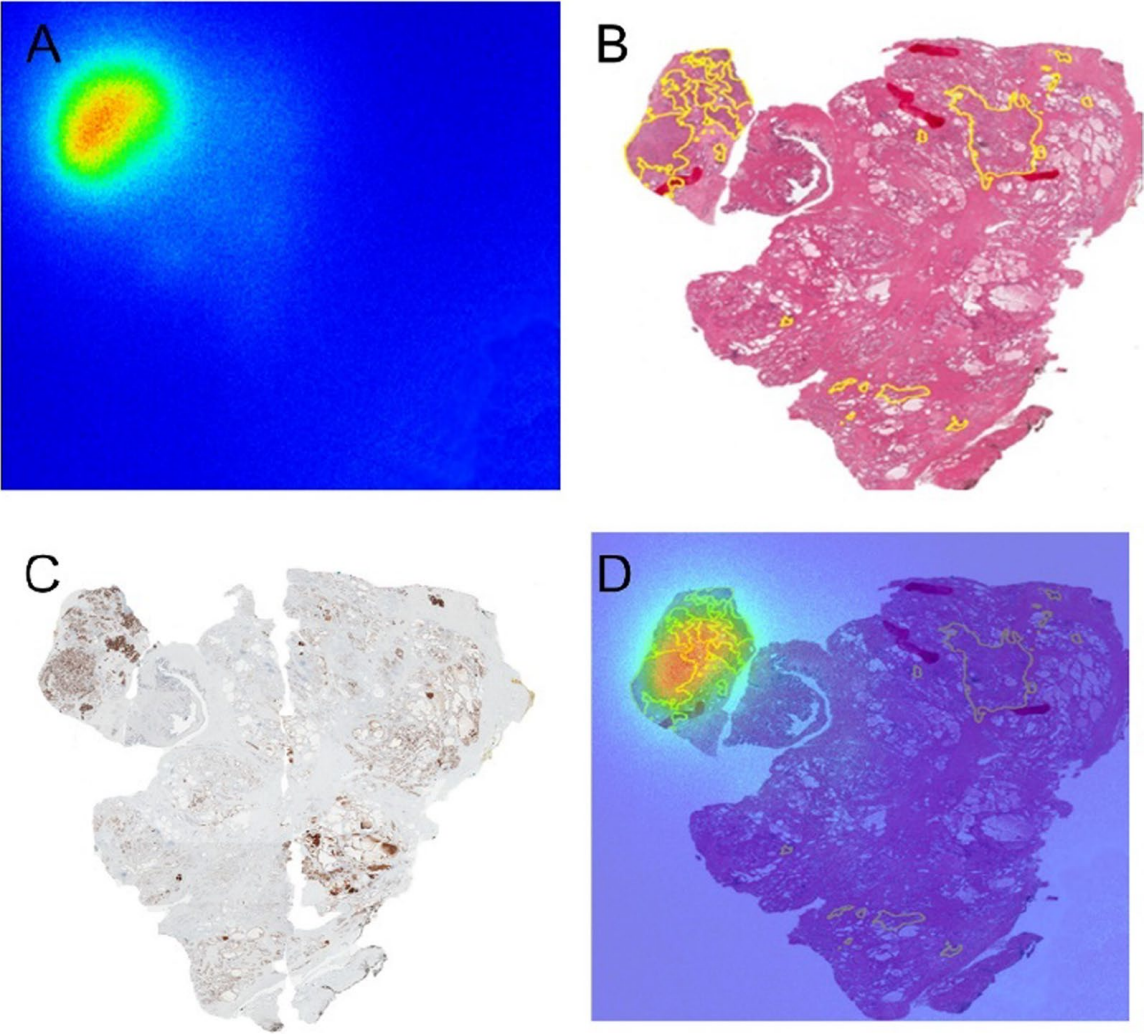
Representative autoradiographic and histopathological images. **A** Autoradiographic image. **B** HE staining image. *Yellow region* prostate cancer. (C) PSMA IHC staining image. (D) Fused image of ARG and HE. ARG = autoradiography, HE = Hematoxylin and Eosin, IHC = immunohistochemistry, PSMA = prostate-specific membrane antigen.

Overall, ARG was performed in 37 prostate slices from 17 patients. A representative setup for ARG is presented in Additional file 1: Fig. 3. ARG and corresponding histopathological and immunohistopathological images are shown in Fig. 1.

### Correlation between PSMA-ligand uptake, histopathology and immunohistochemistry

#### Heterogeneity of PSMA IHC and PSMA-ligand uptake

**Fig. 2:**
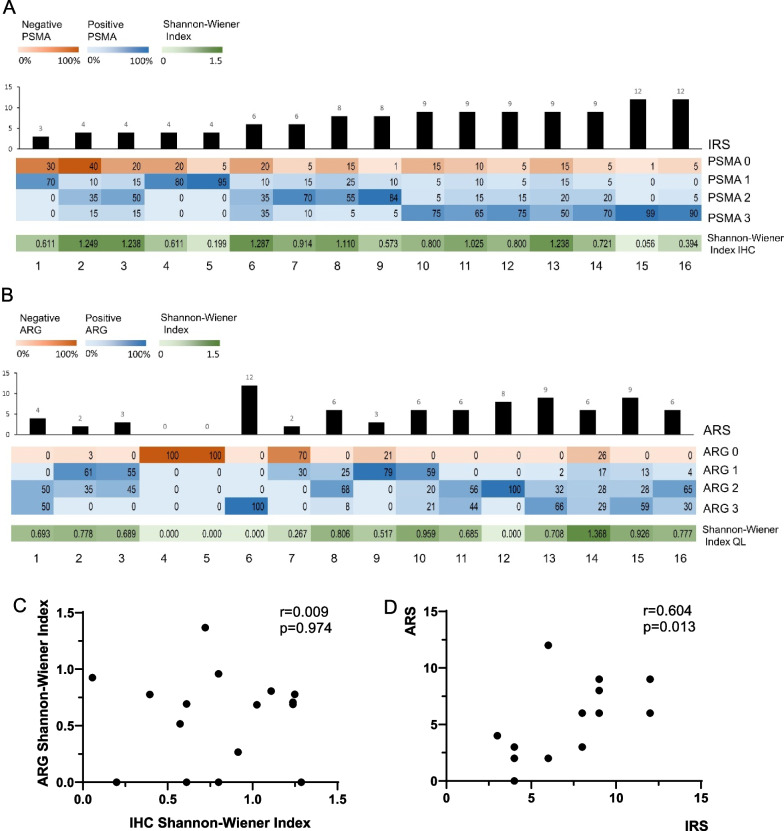
PSMA protein expression and PSMA-ligand uptake are heterogeneous. Expression of PSMA protein quantified by IRS (**A**) was presented in order of increasing score. PSMA-ligand uptake quantified by ARS (**B**) was presented in the corresponding order. Degrees of heterogeneity in PSMA protein expression and PSMA-ligand uptake were measured by Shannon–Wiener Index and depicted as heat map ranging from low heterogeneity (white) to high heterogeneity (green). Correlation between (**C**) Shannon–Wiener Index of ARG and IHC (Pearson, *r* = 0.009, *p* = 0.974), (**D**) ARS and IRS (Pearson, *r* = 0.604, *p* = 0.013). ARS = autoradiographic-reactive score, IHC = immunohistochemistry, IRS = immunoreactive score, PSMA = prostate-specific membrane antigen. Patients were renumbered in the current study: P18 = 1; P10 = 2; P11 = 3; P17 = 4; P20 = 5; P9 = 6; P13 = 7; P12 = 8; P15 = 9; P3 = 10; P7 = 11; P8 = 12; P16 = 13; P19 = 14; P4 = 15; P5 = 16

PSMA IHC was performed in 16 postoperative prostate specimens, one dropped out due to PIN. The median IRS was 8 (interquartile range: 4–9). All 37 tissue sections of the 16 postoperative prostate specimens showed tumor areas without immunohistochemically detectable expression of PSMA (100%). The Median ARS representing the PSMA-ligand uptake levels was 6 (interquartile range: 2.25–7.5). Of 16 samples, the distribution of ARS had a similar trend as IRS. A significant moderate correlation between IRS and ARS was present (*r* = 0.604, *p* = 0.013, Fig. 2D). However, comparing individual IRS and ARS, remarkable differences were observed, with, e.g., P9 showing the highest ARS but only a moderate IRS score.

The Shannon–Wiener index was calculated to quantify the degree of heterogeneity of PSMA IHC and ^99m^Tc-PSMA-I&S uptake. Marked inter-patient and intra-patient heterogeneity was observed. A broad range of the Shannon–Wiener Index for IHC was present (0.056–1.249, Fig. 2A). Only two patients (Patient 5 and 15) showed a low heterogeneity and four patients presented with a relatively high heterogeneity (Shannon–Wiener Index > 1.2, Patient 2, 3, 6, and 13).

A broad range of the Shannon–Wiener Index was also noted for ARG. A homogenous signal (Shannon–Wiener Index of 0.0) was present in 4 patients (Fig. 2B): Two patients exhibited no signal in ARG (Patient 4 and 5) and two patients showed a homogenously moderate (Patient 12) and high (Patient 6) ARG signal. No correlation between the Shannon–Wiener Index for IHC and ARG was observed (*r* = 0.009, *p* = 0.974, Fig. 2C). Of note, the patient with the highest Shannon–Wiener Index in IHC and a moderate IRS score (Patient 6) showed homogeneity in ARG with the highest ARS score.

##### Region based analysis

**Fig. 3:**
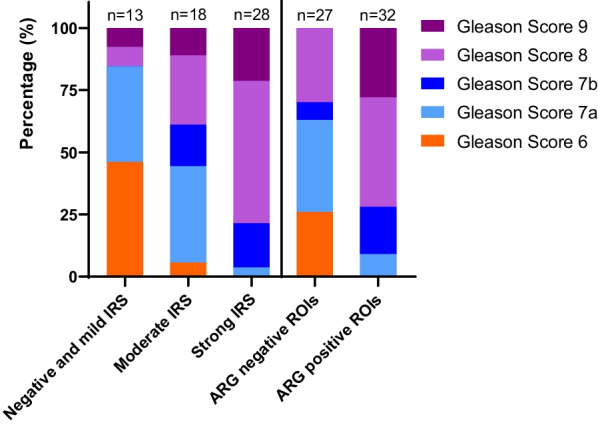
Bar chart of Gleason Scores with PSMA expression and ARG. ARG = autoradiography, PSMA = prostate-specific membrane antigen

**Fig. 4:**
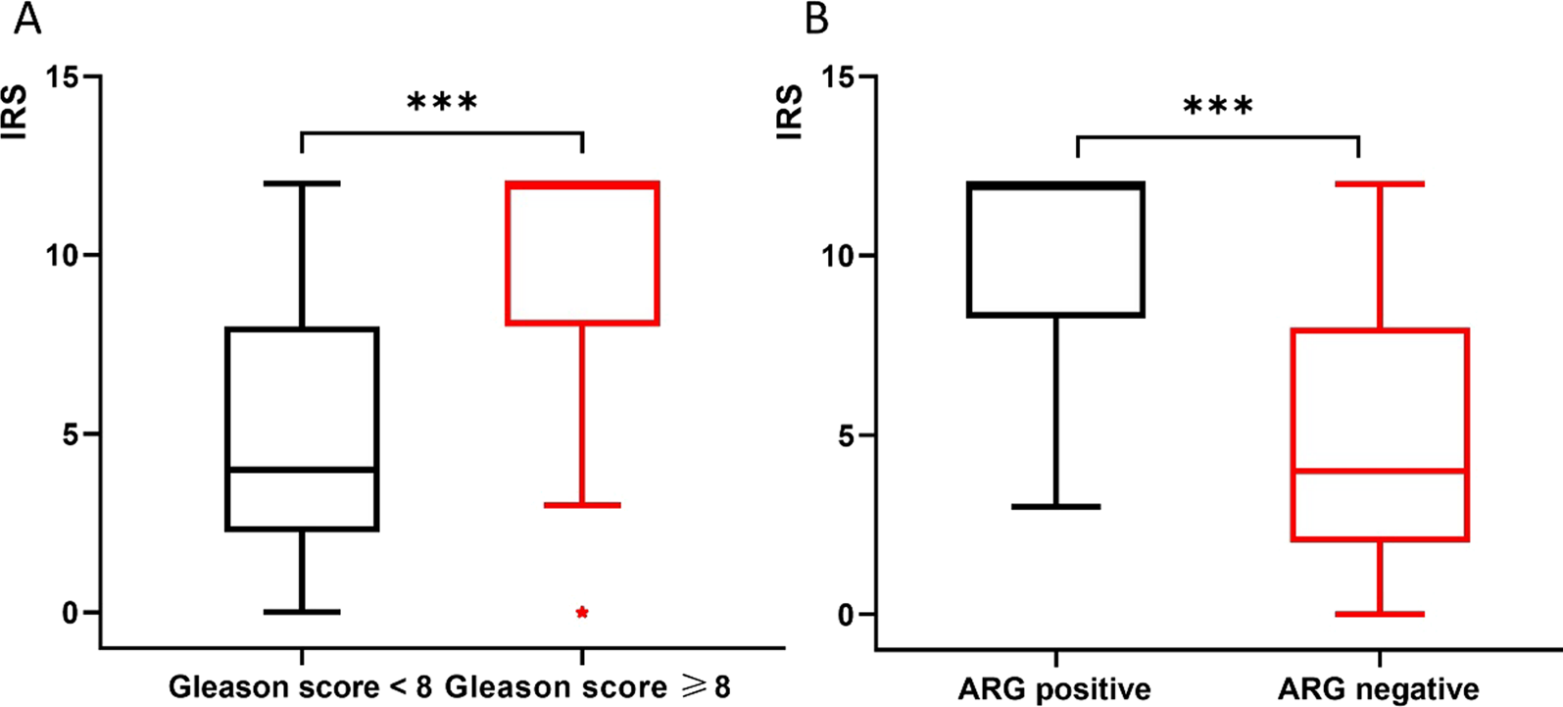
**A** Box plot of the correlation between Gleason Score and IRS. An higher IRS score was observed in Gleason Score ≥ 8 (***Mann–Whitney U test, *p* < 0.001). **B** Box plot indicating a higher IRS score in ROIs with a positive signal in ARG (***Mann–Whitney U test, *p* < 0.001). ARG = autoradiography, IRS = immunoreactive score, ROI = region of interest

A total of 59 ROIs were included in the analysis. Figure 3 shows the percentages of Gleason Scores in different IRS classification. The percentage of patients with low vs. high Gleason Score was clearly related to the signal in IHC and ARG (Fig. 3): Exemplarily in ROIs with negative and mild IRS (*n = *13), 46.1% (*n = *6) were graded as Gleason Score 6, 38.5% (*n = *5) as Gleason Score 7a, 0% (*n = *0) as Gleason Score 7b, 7.7% (*n = *1) as Gleason Score 8 and 7.7% (*n = *1) as Gleason Score 9. In contrast, ROIs with strong IRS (*n = *28), 0% (*n = *0), 3.6% (*n = *1), 17.9% (*n = *5), 57.1% (*n = *16) and 21.4% (*n = *6) were rated as Gleason Score 6, 7a, 7b, 8 and 9, respectively. Median IRS was significantly higher in regions with Gleason Score ≥ 8 (median 4 (IQR 2.25–12.0) vs. 12 (8.0–12.0), *p* < 0.001, Fig. 4A).

In ARG 32 (65.3%) of 59 ROIs showed a positive signal. Positive ROIs in ARG contained Gleason Score 7a, 7b, 8 and 9 in 3 (9%), 6 (19%), 14 (44%) and 9 (28%) tumors in histopathology, respectively. The 27 ROIs with negative signal in ARG contained Gleason Score 6, 7a, 7b and 8 in 7 (26%), 10 (37%), 2 (7%), and 8 (30%) (Fig. 3). The percentage of patients with low vs. high Gleason Score is clearly related to the signal in ARG. Visually positive signal in ARG was associated with a higher median IRS (median 4.0 (2.0–8.0) vs. 12 (8.25–12.0), *p* < 0.001, Fig. 4B).

##### Grid based analysis

**Fig. 5:**
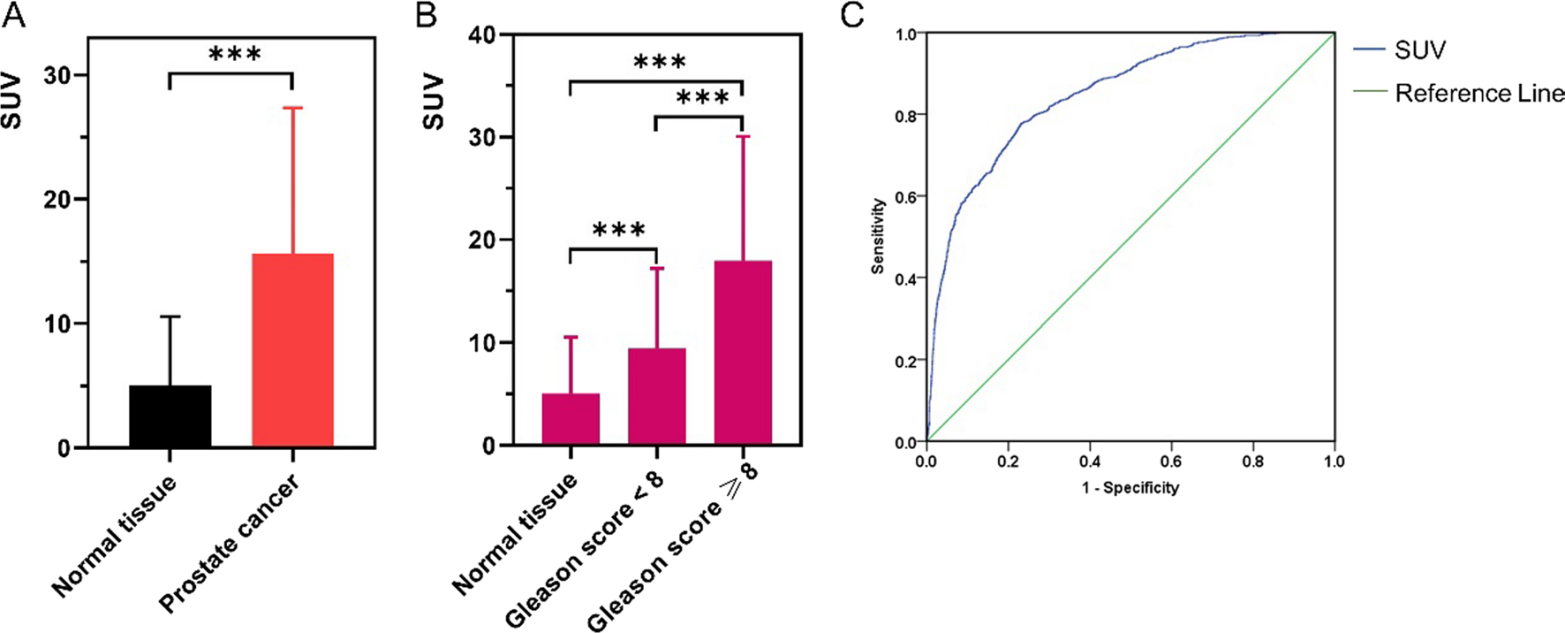
**A** Bar chart for quantitative signal in ARG (SUV_ARG_) for normal tissue and prostate cancer containing grids. High uptake of ^99m^Tc-PSMA was present in grids with prostate cancer (SUV_ARG_, ***Mann–Whitney U test, p < 0.001). **B** Significantly higher SUV_ARG_ was observed in the Gleason Score ≥ 8 compared to normal prostate tissue and Gleason Score < 8 groups (***ANOVA, *p* < 0.001). **C** Receiver-operating-curve analysis. A cutoff of 6.2 for SUV_ARG_ discriminated best between normal tissue and prostate cancer and yields a sensitivity of 77.7% and specificity of 76.8% (area under curve, 0.848). ARG = autoradiography, PSMA = prostate-specific membrane antigen, SUV = standardized uptake value

A total of 4660 grids were included in the grid-based analysis. A significantly higher ^99m^Tc-PSMA-I&S uptake (SUV_ARG_) was observed in areas with prostate cancer compared with benign prostate tissue (15.6 ± 11.7 in *n = *1177 vs. 5.0 ± 5.4 in *n = *3483 grids; *p* < 0.001, Fig. 5A). Both Gleason Score ≥ 8 and Gleason Score < 8 presented with significantly higher SUV_ARG_ compared to normal prostate tissue (17.9 ± 12.1 in *n = *855 and 9.4 ± 7.8 in *n = *322 vs. 5.0 ± 5.4 in *n = *3483 grids, *p* < 0.001, respectively, Fig. 5B).

The AUC for SUV_ARG_ (Fig. 5C) to discriminate between prostate cancer and benign prostate tissue was 0.848 (95% CI 0.835–0.860). The optimal threshold for SUV_ARG_ determined by ROC-analysis was 6.2 and resulted in a sensitivity and specificity of 77.7% and 76.8%, respectively.

Application of the linear mixed model method revealed a significant association between SUV_ARG_ and the presence of GP 4, GP 5, PIN, and stroma (*p* < 0.001, *p* = 0.013, *p* = 0.035, *p* = 0.002, respectively; Table 3). The results demonstrated that there was a 0.43% increase in SUV_ARG_ for every 1% increase in GP 4, and there was a 0.07% increase in the PSMA-ligand uptake for every 1% increase in GP 5.

**Table 3:** PSMA-ligand uptake correlates with aggressive malignancy

Variable	Coefficient	*p* value	95% confidence interval	
			Lower bound	Upper bound
SUV				
GP 3	− 0.10	0.093	− 0.22	0.02
GP 4	0.43	0.000	0.41	0.46
GP 5	0.07	0.013	0.01	0.12
Normal epithelium	− 0.01	0.611	− 0.05	0.03
Seminal Vesicle	0.15	0.078	− 0.02	0.31
PIN	0.24	0.035	0.02	0.47
Inflammation cells	− 0.05	0.177	− 0.11	0.02
Stroma	0.02	0.002	0.01	0.02

GP, Gleason pattern; PIN, prostatic intraepithelial neoplasia; SUV, standardized uptake value

## Discussion

PSMA is an established target for theranostics of prostate cancer [43], and recently it has drawn increasing interest. Prior work has demonstrated that the intensity of PSMA expression can serve for risk stratification. A study from Ross et al. indicated that patients with tumors that show high PSMA expression in immunohistochemistry had a significantly increased rate of tumor grade (*p* = 0.03), pathological stage (*p* = 0.029), aneuploidy (*p* = 0.01) and biochemical recurrence (*p* = 0.001) as compared to tumors featuring a relatively lower PSMA expression [13].

Moreover, PSMA negative primary tumor limiting the potential of PSMA PET to detect its metastases and showing insufficient response to PSMA-targeted radionuclide therapy have been described in literature [17, 44]. Whether this is related to a uniform PSMA-negative cancer infiltrates or mosaics of missing and high PSMA expression in tumor deposits leading to low imaging signal and missing response to treatment is not yet fully understood. PSMA-ligands and antibodies have different size and binding position, thus, research comparing PSMA PET and IHC is prone to methodological limitations. Our aim was to describe patterns of ^99m^Tc-PSMA-I&S uptake and correlate the respective autoradiography signal with Gleason Grading and (immuno)-histopathology [45]. A further intriguing aspect of this approach is that the signal of radioactivity arises from the exact same tissue, which is further processed for histopathology sections. In this way, the misalignment caused by manual image rotation and processing was avoided and we could then investigate the tracer uptake on nearly microscopic level.

In our study, we observed marked intra-patient heterogeneity as assessed by the Shannon–Wiener index. High inter-patient heterogeneity, as expressed by the IRS and ARS, was also observed. Signals from IRS and ARS showed a moderate correlation (*r* = 0.604; *p* = 0.013). The strength of our study arises from the direct comparison between the ^99m^Tc-PSMA-I&S signal in ARG, histopathology (Gleason grading) and PSMA IHC of exactly the same tissue. To the best of our knowledge, our study is the first to evaluate this using specific grid-based annotation and high-resolution ARG.

Our results indicate a considerably higher signal in ARG in Gleason Score ≥ 7b prostate cancer tissues (Gleason Score ≥ 7b prostate cancer ROIs with ARS positive and ARS negative: 29 (91%) and 10 (37%), respectively). In line with our findings, a study that included 74 patients indicated that PSMA-negative tumor areas negatively correlated with Gleason Score [46]. Moreover, our data suggest that prostate cancer is a heterogeneous disease, and intratumor heterogeneity in IHC has no correlation with heterogeneous ^99m^Tc-PSMA-I&S uptake. Similarly, Paschalis et al. reported the heterogeneous expression of PSMA and observed that the degree of heterogeneity was positively correlated with the PSMA expression level [14]. The current investigation is the first attempt to develop a semi-quantification system of ^99m^Tc-PSMA-I&S uptake in parallel to systems established in histopathology. Specifically, we introduced an ARS system to assess the uptake of ^99m^Tc-PSMA-I&S, which includes both signal area and intensity. Moreover, the heterogeneity was analyzed quantitatively using Shannon–Wiener Index.

Multiple investigations have demonstrated that the uptake of radiolabeled PSMA-ligands in prostate cancer is higher than in normal prostate tissue. However, the threshold of SUV varies depending on the study. Rahbar et al. reported a significant difference (*p* < 0.001) in median SUV_max_ between true-positive prostate cancer (11.0 ± 7.8) and normal prostate tissue (2.7 ± 0.9) using ^68^ Ga-PSMA-HBED-CC as radioligand [47]. Furthermore, Woythal et al. documented a significantly higher SUV_max_ of prostate cancer (14.06 ± 15.56) than that of normal prostate (2.43 ± 0.63; *p* < 0.001). In line with these results, our results indicate a significantly higher uptake of ^99m^Tc-PSMA-I&S in malignant lesions than in cancer-free prostate tissue. Being the first group investigating PSMA-ligand autoradiography our data propose a cutoff SUV_ARG_ for predicting prostate cancer from non-tumor tissue using ^99m^Tc-PSMA ARG data. Although results from ARG are not robust evidence for generating a cutoff for PSMA-ligand PET because of the variety of ligands and calculation procedure, it still can be an alternative reference. More studies are now needed to determine the association between SUV_max_ and SUV_ARG_.

We have analyzed the correlation between ^99m^Tc-PSMA-I&S uptake and Gleason Patterns, in order to demonstrate the correlation between tracer distribution and clinicopathology and to study the intraprostatic tracer uptake at microscopic level. Bravaccini et al. observed stronger PSMA staining intensity in GP 4 and 5 than in GP 3, and Woythal et al. reported that the SUV_max_ in ^68^ Ga-PSMA-11 PET correlated with PSMA expression in primary prostate cancer [38]. Similarly, our results indicate that prostate cancer with GP 4 and 5 correlated with a significantly higher tracer uptake compared with non-neoplastic prostate tissue (*p* < 0.001, *p* = 0.013, respectively). Although the appearance of PIN and stroma significantly correlated with ^99m^Tc-PSMA-I&S uptake (*p* = 0.035, *p* = 0.002, respectively), the results should be interpreted with caution. The sample size in PIN group is relatively small. Our results highlight the promising role of radiolabeled PSMA-ligands in the prediction of tumor aggressiveness and extend its use to ARG.

The present study has several limitations. First of all, our results are limited by the retrospective nature of the study and the relatively small size of the patient cohort. Besides, although the autoradiographic and histopathological images come from the same tissue, the sample thickness for ARG is around 5 mm, and the thickness of samples for histopathology is 2 µm. Moreover, in the current study, we included over 4000 grids in the analysis. However, the sample size varies in each subgroup. Thus, further studies with more tumor samples are needed. The time interval between tracer injection and ARG was over 18 h which could induce a bias due to decay especially when lesions / grids are only showing low initial PSMA-ligand uptake. Finally, tissue configuration shrinkage happens during histological sample preparation, which might cause misalignment during imaging registration. Although we performed ARG that has a higher resolution compared with clinical PET images, new approaches are still needed for further studies.

## Conclusion

We evaluated the intraprostatic ^99m^Tc-PSMA-I&S distribution using high-resolution ARG. Heterogeneous expression of PSMA and tracer uptake were observed. Intraprostatic ^99m^Tc-PSMA-I&S uptake was associated with PSMA expression, Gleason Score and Gleason Pattern, with high ^99m^Tc-PSMA-I&S uptake in ARG being observed in higher grade tumors. Our study underlines the positive association between PSMA expression and the uptake of radiolabeled PSMA-ligands and extends its use to ARG.

### Supplementary Information

**Additional file 1. Fig. S1.** Sample preparation. **A**: The prostate specimen from RP (black lines indicate the resected parts for cryosections). **B**: The prostate specimen was cut from base to apex and perpendicular to the long axis of the urethra. **C**: Every 2nd slice of the specimen was fixed in a sealed plastic bag filled with 10% neutral-buffered formalin for ARG. **Fig. S2.** Schematic diagram of ARG for in vivo PSMA-ligand uptake analysis. **Fig. S3.** Representative tissue samples for ARG. *Black star* standards.

## Data Availability

The datasets used and/or analyzed during the current study are available from the corresponding author on reasonable request.

## Abbreviations

^177^Lu: Lutetium-177
^99m^Tc: Technetium-99 m
ARG: Autoradiography;
AUC: Area under the curve
CEP: Comparative experimental pathology
DAB: Diaminobenzidine
FFPE: Formalin fixed paraffin embedded
GP: Gleason pattern
GS: Gleason score
HE: Hematoxylin and eosin
IHC: Immunohistochemistry
IQR: Interquartile range
IRS: Immunoreactive score
ISUP: International society of urologic pathologists
PET: Positron emission tomography
PIN: Prostatic intraepithelial neoplasia
PSMA: Prostate-specific membrane antigen
QL: Quantum level
RGS: Radioguided surgery
RLT: Radioligand therapy
ROC: Receiver operating characteristic
ROI: Region of interest
SD: Standard deviation
SDI: Shannon's diversity index
SUV: Standardized uptake value
TUM: Technical University of Munich
