# Supplementary material for: Heterogeneity of prostate-specific membrane antigen (PSMA) and PSMA-ligand uptake detection combining autoradiography and postoperative pathology in primary prostate cancer
Source: EJNMMI Res. 2023 Nov 16;13:99. doi: 10.1186/s13550-023-01044-8 (PMC10654338; doi:10.1186/s13550-023-01044-8)

Supplementary Fig. 1. Sample preparation.

A: The prostate specimen from RP (black lines indicate the resected parts for cryosections). B: The prostate specimen was cut from base to apex and perpendicular to the long axis of the urethra. C: Every 2^nd^ slice of the specimen was fixed in a sealed plastic bag filled with 10% neutral-buffered formalin for ARG.


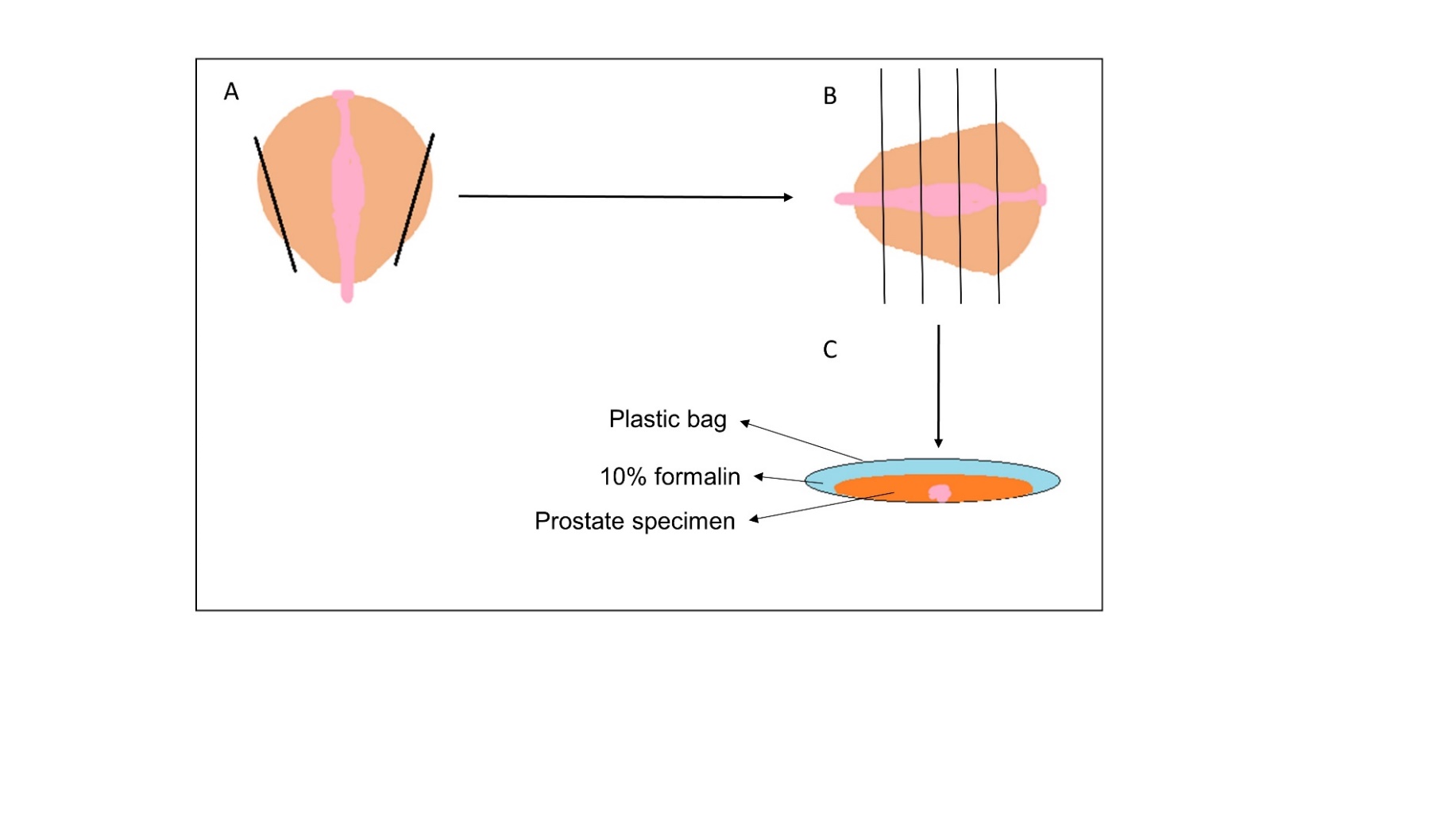


Supplementary Fig. 2. Schematic diagram of ARG for in vivo PSMA-ligand uptake analysis.


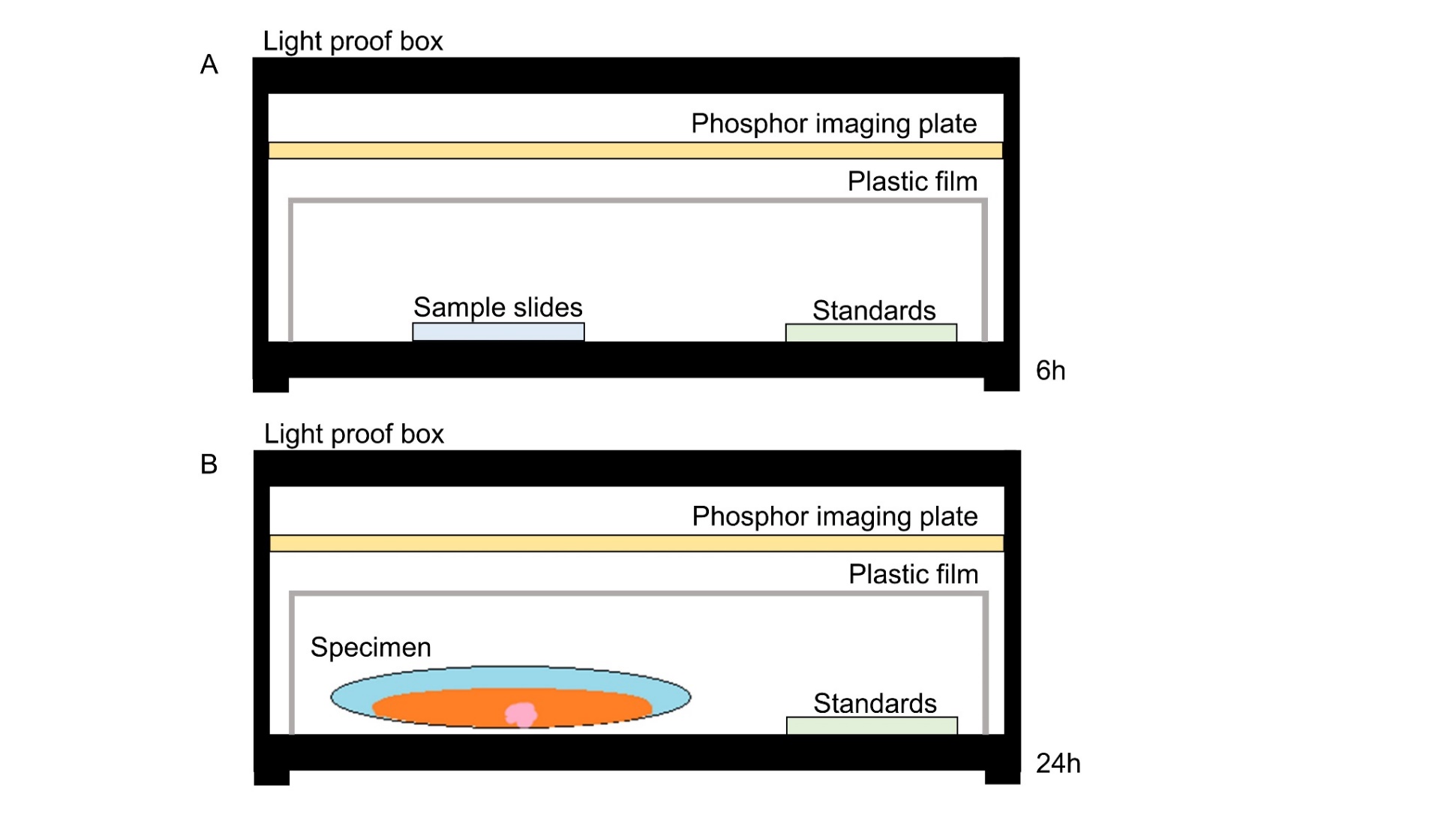


Supplementary Fig. 3. Representative tissue samples for ARG. *Black star* standards.


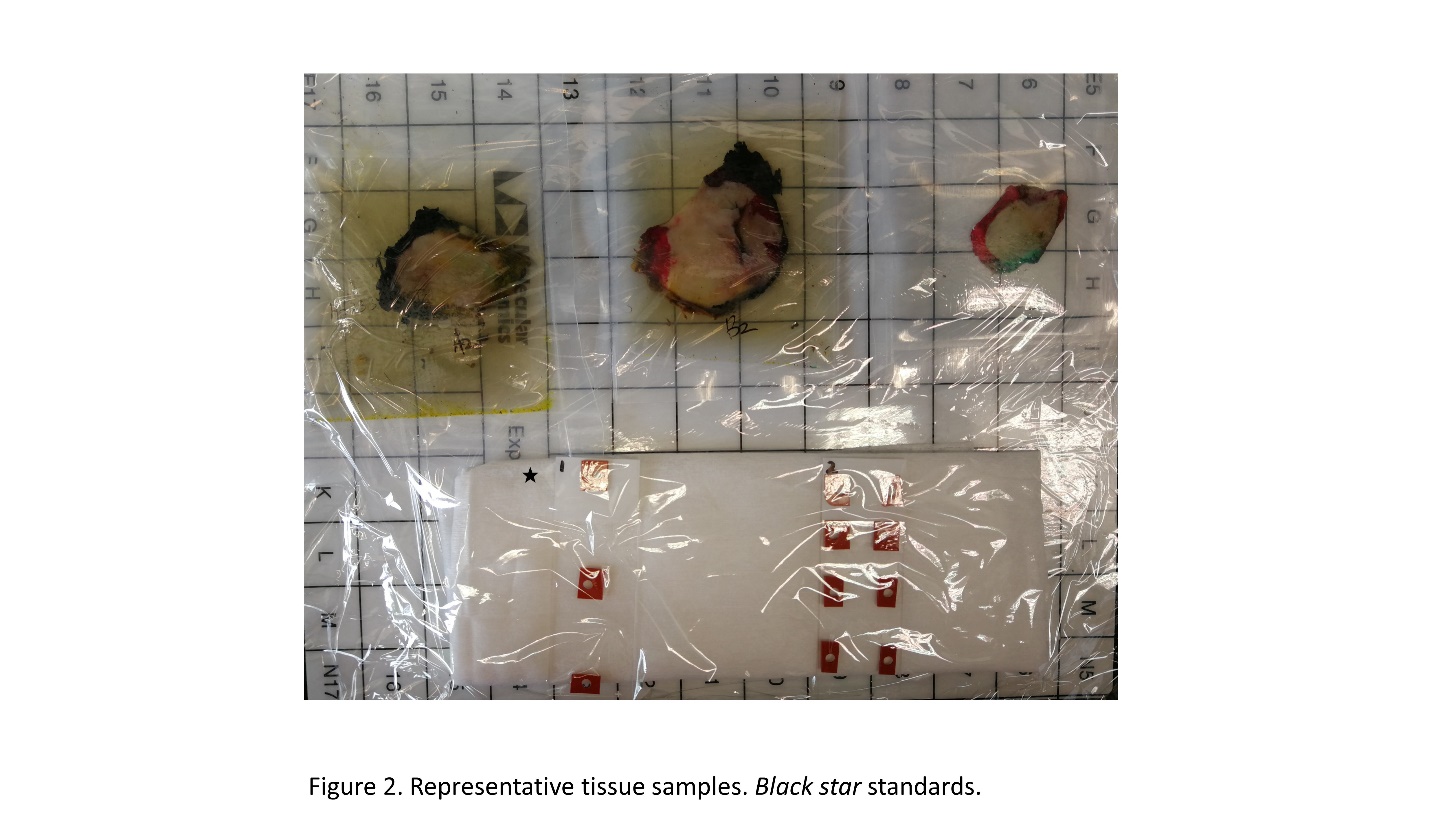

Supplement: Supplementary file 1 — Additional file 1. Fig. S1. Sample preparation. A: The prostate specimen from RP (black lines indicate the resected parts for cryosections). B: The prostate specimen was cut from base to apex and perpendicular to the long axis of the urethra. C: Every 2nd slice of the specimen was fixed in a sealed plastic bag filled with 10% neutral-buffered formalin for ARG. Fig. S2. Schematic diagram of ARG for in vivo PSMA-ligand uptake analysis. Fig. S3. Representative tissue samples for ARG. Black star standards. [file 13550_2023_1044_MOESM1_ESM.docx]
